# Lumpectomy followed by radiation improves survival in HER2 positive and triple‐negative breast cancer with high tumor‐infiltrating lymphocytes compared to mastectomy alone

**DOI:** 10.1002/cam4.4050

**Published:** 2021-06-03

**Authors:** Jason A. Mouabbi, Momal Chand, Ishaq A. Asghar, Ramen Sakhi, Daniel Ockner, Carrie L. Dul, Tarik Hadid, Amr Aref, Mothaffar F. Rimawi, Valentina Hoyos

**Affiliations:** ^1^ Dan L Duncan Comprehensive Cancer Center Baylor College of Medicine Houston TX USA; ^2^ Ascension St John Hospital Detroit MI USA

**Keywords:** breast cancer, HER2+, radiation therapy, TILs, TNBC, triple‐negative, tumor‐infiltrating lymphocytes

## Abstract

**Objective:**

The goal was to compare the 5‐year DFS and 5‐year OS in patients with early‐stage human epidermal growth factor receptor 2 breast cancer (HER2+ BC) and triple‐negative breast cancer (TNBC) in relation to the amount of stromal tumor‐infiltrating lymphocytes (TILs) after locoregional management by either mastectomy without radiation or lumpectomy and whole‐breast radiotherapy (RT).

**Methods:**

This was a retrospective review of HER2+ BC and TNBC patients’ charts and histopathology slides with clinical stage of T1‐T2 N0 who presented at our facility between January 2009 and December 2019. Locoregional treatment included either mastectomy without RT (M) or lumpectomy with RT (L+R). TILs were assessed by three pathologists using the guidelines of the 2014 TILs working group. A competing risk model and Kaplan–Meier analysis were used to analyze correlations between TILs levels and clinical outcome.

**Results:**

We reviewed 211 patients’ charts. Of them, 190 proceeded to the final analysis. Patients were split into groups of "low TILs" and "high TILs" based on a 50% TILs cut‐off. Of them 26% had high TILs, 48% received RT, 97% received chemotherapy, all HER2+ BC patients received HER2‐directed therapy and all HER2+ BC that were also hormone receptor positive (HR+) received endocrine therapy (ET). In patient with low TILs, L+R did not improve outcomes compared to M. Moreover, patients with high TILs had a significant improvement of their DFS and OS with L+R when compared to M.

**Conclusion:**

The results of our study reflect that a selected group of HER2+ BC and TNBC with elevated TILs, L+R is associated with improvement of 5‐year DFS and 5‐year OS.

## Graphical abstract

A quarter of HER2+ and triple‐negative breast cancer patients have high levels of tumor‐infiltrating lymphocytes. HER2+ and triple‐negative breast cancer patients have high levels of tumor‐infiltrating lymphocytes have improved 5‐year DFS and OS if they receive radiation as part of their locoregional therapy.

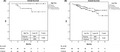

## 1 INTRODUCTION

Immune system recognizes transformed malignant cells and inhibits their growth through immunosurveillance mechanisms.^1^, ^2^ Tumor‐infiltrating lymphocytes (TILs) represent a surrogate marker for this anti‐tumor immune response.^3^

Around 11% of breast cancers demonstrate high levels of TILs. Human epidermal growth factor receptor 2 positive breast cancers (HER2+ BC) and triple‐negative breast cancer (TNBC) demonstrated the highest incidence around 20%.^4^

Clinically, HER2+ BC and TNBC are aggressive subtypes of breast cancer, exhibiting a high rate of cancer cell proliferation. This is correlated with increased genomic instability and antigenicity. In fact, HER2+ BC and TNBC have a 13‐fold higher rate of mutations over hormone receptor positive (HR+), HER2 negative breast cancers.^5^ These tumor‐associated mutations result in the generation of neo‐antigens, which can be strong immunogenic targets that elicit potent antitumor T cell responses.^6^, ^7^

Increased levels of TILs in women receiving neoadjuvant or adjuvant chemotherapy are associated with improved recurrence‐free and overall survival (OS) in HER2+ BC and TNBC.^8^, ^9^, ^10^, ^11^, ^12^, ^13^, ^14^, ^15^, ^16^

In the 1970s, a landmark study (NSABP B06) randomized women with stage I or II invasive breast cancer to either undergo lumpectomy, lumpectomy and radiation therapy (L+R), or total mastectomy (M). In 2002, a 20‐year follow‐up showed that there is no difference in survival between women who underwent L+R compared to M. Furthermore, treatment with L+R reduced the rate of recurrence in the ipsilateral breast compared to lumpectomy alone or M.^17^

Recently, two studies done by The Danish Breast Cancer Group (DBCG) and The Swedish Breast Cancer Group showed conflicting results when looking at the interaction of RT with TILs. The Danish group looked at the interaction of TILs with RT in patients who underwent mastectomy. A high TIL level (>30%) was associated with improved OS and decreased risk of distant recurrence. In contrast to that, the Swedish group looked at the interaction of TILs with RT in patients who underwent lumpectomy. In this study, RT was significantly beneficial in the low TIL group (<10%) but not in the higher TIL group (>10%). ^18^, ^19^

Given the conflicting data from the two aforementioned studies, more data are needed in this area. We conducted a retrospective analysis with the purpose of exploring outcomes in patients with early‐stage HER2+ BC and TNBC in relation to TILs levels after locoregional management by either M or L+R.

## 2 METHODS

### 2.1 Study population and study design

This study is a retrospective review of medical records and pathology samples of all patients with early‐stage (defined as T1‐T2 N0 disease) HER2+ BC and TNBC who underwent locoregional management from January 2009 to December 2019 at Ascension St John Hospital in Detroit, Michigan. Locoregional management included either mastectomy without radiation (M) or lumpectomy with radiation (L+R).

### 2.2 Analysis of tissue samples

Formalin‐fixed, paraffin‐embedded (FFPE) tissue slides that are hematoxylin‐eosin (HE) stained were reviewed by three pathologists. To quantify TILs, we followed the guidelines published by the TILs working group in 2014.^20^

Briefly, TILs include both lymphocytes and plasma cells. Based on the TILs working group, only stromal TILs within the boundaries of the invasive tumor are evaluated. Parts that contain crush artifacts, necrosis, and previous core biopsy sites are excluded. The estimation is reported as a percentage of the area of stromal tissue occupied by TILs compared to the total area of intratumoral stroma. TILs were categorized into low TILs and high TILs based on a 50% cut‐off.^20^

### 2.3 Statistical analysis

Descriptive statistics were generated to characterize the study group. Continuous variables were described as median with range.

The endpoints of interest were the 5‐year DFS, and OS. We defined 5‐year DFS as the percentage of patients that did not relapse (local or distant) from the time of disease diagnosis; and 5‐year OS as the percentage of patients that did not die, irrespective of cause, from the time of disease diagnosis.

We assessed correlations between TILs, radiation therapy, age, tumor stage, and chemotherapy by χ² test or Fisher's exact tests, and by logistic regression; correlations with 5‐year DFS and OS were assessed by Cox proportional hazard regression.

All data were analyzed with SPSS v. 25.0 and a *p* value of 0.05 or less was considered statistically significant.

## 3 RESULTS

**FIGURE 1:**
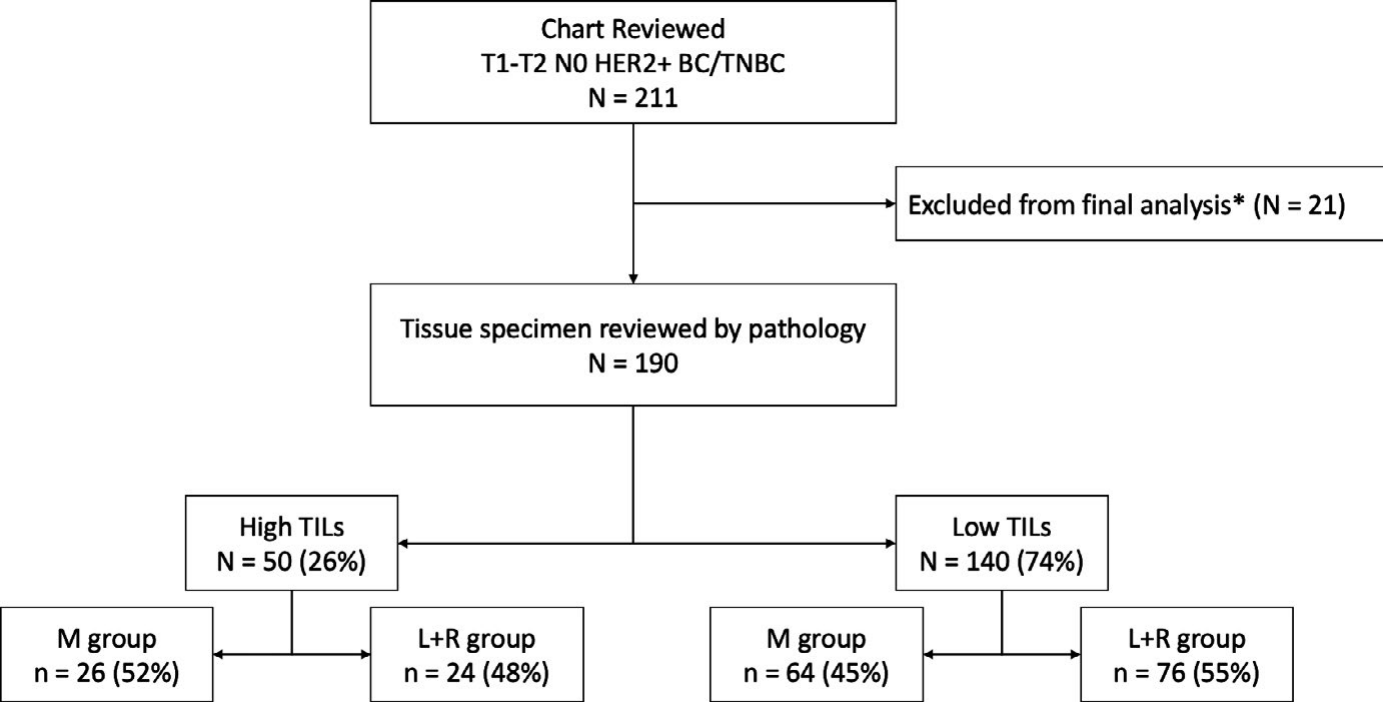
CONSORT Diagram. HER2+ BC, Human epidermal growth factor receptor 2 positive breast cancer; TNBC, triple‐negative breast cancer; TILs, tumor‐infiltrating lymphocytes; RT, radiation therapy; *21 patients excluded due to non‐availability of pathology specimen

We reviewed 211 patients’ charts. Of them, 190 proceeded to the final analysis. Of them, 26% (*n* = 50) had high TILs. Around 50% of patients in the high TILs (*n* = 24) and low TILs (*n* = 75) groups received RT (Figure 1).

Table 1 shows the characteristics of the study population. The median age was around 59 years and most of the patients were Caucasian (76%). Half the patients had HER2+ and the other half had TNBC. The majority (70%) were deemed HER2+ by having an intense circumferential HER2 immunohistochemistry stain (IHC 3+). Around 70% of HER2+ patients were also HR+. Patients were split almost equally between stages T1N0 and T2N0. Almost all patients across all groups received chemotherapy (96%–100%) and approximately half of them received it in the neoadjuvant setting. All HER2+ BC patients received HER2‐directed therapy and all HR+patients received endocrine therapy in the adjuvant setting.

**TABLE 1:** Patient characteristics

	High TILs *N* = 50		*p* value	Low TILs *N* = 140		*p* value
	M group *N* = 26	L+R group *N* = 24		M group *N* = 64	L+R group *N* = 76	
Age (Median) — yr. (range)	57 (38 – 80)	58 (40 – 81)	0.553	60 (31 – 89)	61 (31 – 85)	0.100
Race — no. (%)						
Caucasian	21 (80)	20 (83)	0.950	52 (80)	53 (70)	0.117
African‐American	5 (20)	4 (17)		12 (20)	23 (30)	
Receptor status						
HER2+/HR+	7 (27)	9 (37)	0.564	26 (40)	30 (40)	0.648
HER2+/HR‐	6 (23)	4 (17)		6 (10)	11 (15)	
HER2‐/HR‐	13 (50)	11 (46)		32 (50)	35 (45)	
Stage						
T1 N0	11 (45)	14 (54)	0.316	35 (55)	38 (50)	0.580
T2 N0	15 (55)	10 (46)		29 (45)	38 (50)	
Chemotherapy — no. (%)	26 (100)	23 (96)	0.302	62 (97)	74 (97)	0.861
Neoadjuvant	14 (54)	12 (50)	0.386	29 (45)	38 (51)	0.595
Adjuvant	12 (46)	12 (50)		33 (55)	36 (49)	
HER2+ patients who received HER2‐directed therapy	13 (100)	13 (100)		32 (100)	41 (100)	
HER2+/HR+patients who received endocrine therapy	7 (100)	9 (100)		26 (100)	30 (100)	
Recurrence — no. (%)	6 (23)	0	0.021**	9 (14)	8 (10)	0.715
Local	1 (4)	0	0.322	2 (3)	0	0.121
Distant	5 (19)	0	0.010**	7 (11)	8 (10)	0.938

TILs, tumor‐infiltrating lymphocytes; M, mastectomy; L+R, lumpectomy followed by whole‐breast irradiation; yr, year; no, number; HER2, human epidermal growth factor receptor 2; HR, hormone receptor; **statistically significant *p* value.

In patients with high TILs, there were no recurrences observed in the L+R group, whereas 23% (*n* = 6) of patients in the M group had a recurrence of their disease and most recurrences were distant. Moreover, in patients with low TILs, there was 10% (*n* = 8) recurrence in the L+R group versus 14% (*n* = 9) recurrence in the M group.

**FIGURE 2:**
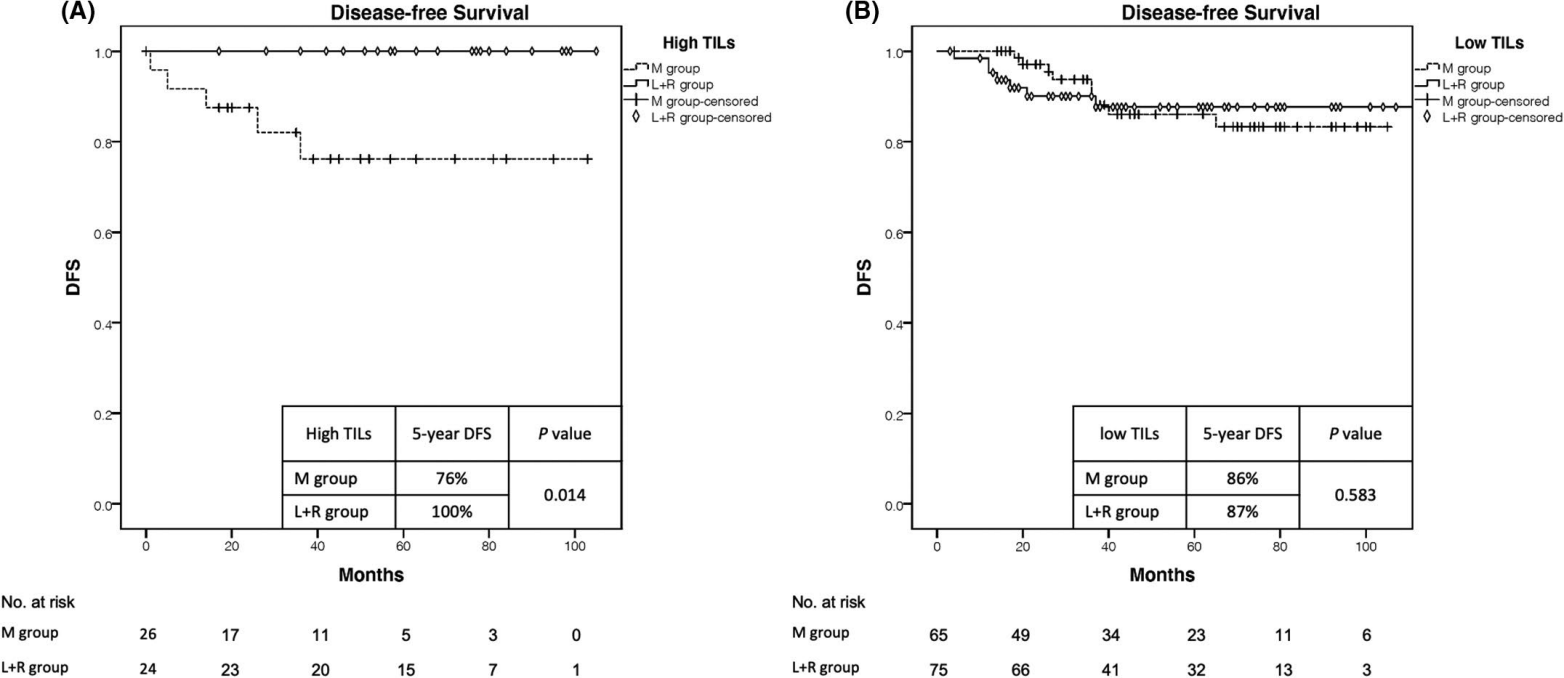
Disease‐free Survival in High TILs (A) and Low TILs (B) groups. DFS, disease‐free survival; TILs, tumor‐infiltrating lymphocytes; M, mastectomy; L+R, lumpectomy followed by whole‐breast irradiation

The 5‐year DFS in the patients with high TILs was 100% in the L+R group whereas it was only 76% in the M group (*p* = 0.014). In the low TILs group, regardless if RT was given, there was no difference in the 5‐year DFS (86% vs 87% in the M and L+R groups, respectively; *p* = 0.583). (Figure 2).

**FIGURE 3:**
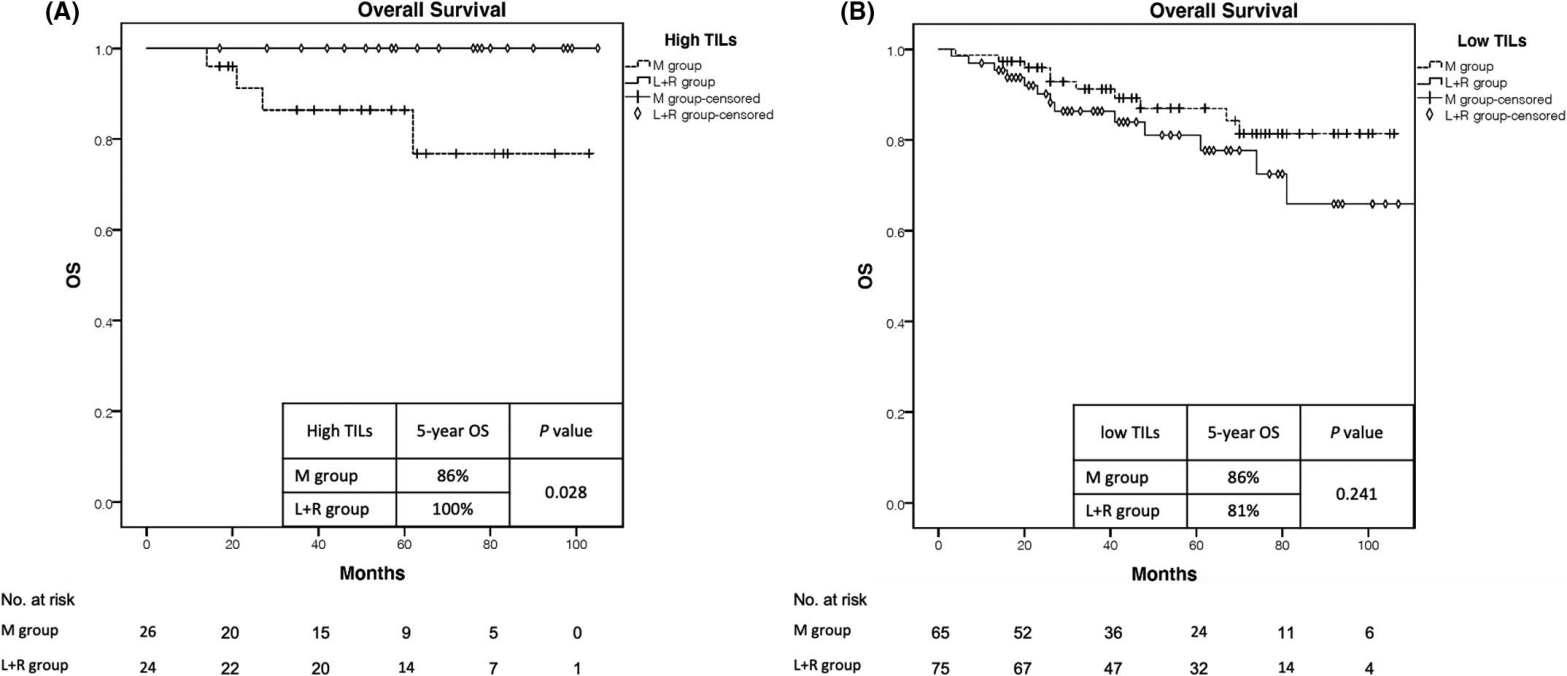
Overall Survival in high TILs (A) and low TILs (B) groups. OS, overall survival; TILs, tumor‐infiltrating lymphocytes; M, mastectomy; L+R, lumpectomy followed by whole‐breast irradiation

The 5‐year OS in the patients with high TILs was 100% in the L+R group whereas it was 86% in the M group (*p* = 0.028). The 5‐year OS difference between the two groups with low TILs was not statistically significant (86% vs 81% in the M and L+R groups, respectively; *p* = 0.241). (Figure 3).

## 4 DISCUSSION

Previous studies on TILs and response to treatment in breast cancer have been inconsistent.^18^, ^19^ The results of our study reflect that in a selected group of HER2+ BC and TNBC with elevated TILs, L+R is associated with improvement of 5‐year DFS and 5‐year OS. This improvement is not likely due to improved locoregional control as local failures were infrequent. Our results support the findings reported by the Danish group rather than the findings of the Swedish breast cancer group.^18^, ^19^

When compared to the Swedish group study, some of the different results might be due to the difference in TILs cut‐off threshold used to define the population between the two studies (10% vs 50%).

The addition of RT after lumpectomy is usually deployed in breast cancer to reduce local recurrence following surgery. Our study did, indeed, show excellent local disease control with no local recurrences observed over a period of 10 years in the patients who received L+R regardless of their TILs level. However, the distant failure was seen in the M group regardless of TILs level and in the L+R group with low TILs might indicate that RT interacts positively with TILs to improve systemic disease control in addition to local disease control. Although our study was not designed to explain the mechanism of interaction between RT and the immune system, it is plausible that RT through tumor damage and release of tumor antigens triggers a local immune response that leads to a systemic effect outside the treatment field. This phenomenon is recognized as the abscopal effect.^21^, ^22^, ^23^, ^24^, ^25^, ^26^, ^27^

Our data are limited due to the fact that the study is retrospective in nature and have a relatively small number of patients with high TILs (50 patients). However, if these results were to be validated in prospective randomized trials, then the now 40‐year‐old concept of the equivalence of M to L+R would be challenged. Although this concept might be true for most patients, however, in a select group of HER2+ BC and TNBC patients who have high TILs, the omission of RT could result in worse outcomes.

## 5 CONCLUSION

In conclusion, high levels of TILs can interact positively with RT to improve outcomes in early‐stage HER2+ BC and TNBC. Our study is limited by its retrospective nature and relatively narrow number of high TILs subjects. Despite that, our results are important and support a personalized approach to the locoregional management of breast cancer, informed by tumor biology, a concept that warrants further study.

## 6 ETHICAL APPROVAL STATEMENT

The ethical approval was from the local IRB at Ascension St John Hospital. This retrospective review of patient's charts and pathological slides was conducted in accordance with the Declaration of Helsinki. The collection and evaluation of all protected patient health information were performed in a Health Insurance Portability and Accountability Act (HIPAA)‐compliant manner.

## CONFLICTS OF INTEREST

NONE

## AUTHOR CONTRIBUTIONS

Dr Jason Mouabbi, Dr Rimawi and Dr Hoyos came up with the concept and design. Dr Mouabbi wrote the study protocol and the current manuscript. Dr Jason Mouabbi, Dr Hadid, and Dr Dul were responsible for data collection. Dr Chand, Dr Asghar, and Dr Shakhi are pathology residents that reviewed the pathology slides and documents the TILs level. Dr Ockner is the pathology attending that supervised the work of Dr Chand, Dr Asghar, and Dr Shakhi. Dr Aref, Dr Rimawi, and Dr Hoyos revised the manuscript and helped with the interpretation of data.

## Data Availability

Data available on request to the corresponding author at the following email: Jason.mouabbi@bcm.edu
